# Political polarization of trust in science

**DOI:** 10.1177/09636625261458088

**Published:** 2026-07-08

**Authors:** Byron Hyde

**Affiliations:** 1 Centre for Ethics in Medicine, Department of Population Health Sciences, Bristol Medical School, UK; 2 School of History, Law and Social Sciences, Bangor University, UK

**Keywords:** public trust in science, public understanding of science, conflicts of interest, politicization of science, political polarization, science communication, science education, science and society

## Abstract

Contrary to widespread claims of a crisis in public trust in science, this article argues that recent pandemic-era trends reflect a long-term pattern of bilateral political polarization rather than a universal decline in scientific confidence. Recent empirical data show that trust and distrust in science have both increased – producing an apparent “pandemic paradox” – driven by ideological bias operating within gaps in public understanding. The study identifies a misunderstanding of how science works as the fundamental cause of politicized trust, where misconceptions create space for ideological bias to shape which claims individuals find credible. Critically, the article argues that existing interventions – improved science communication and increased science education – are insufficient. The core problem is not the quantity of science education but its type. Only by teaching the methodological and institutional limitations of science can the field depolarize public trust and build politically stable confidence in scientific institutions.

## Introduction

Since the pandemic, theorists and policymakers have widely come to believe that there’s a “crisis of trust in science” and are looking for ways to bolster public confidence in institutions. However, recent studies report *increases* in trust. As contradictory evidence emerges, many theorists now maintain that there’s what I call a “pandemic paradox” in which the pandemic fostered both trust and distrust.

In this short essay, I deny that there’s a new crisis of trust in science and argue instead that there’s a long-term trend of *bilateral* polarization caused by ideological bias and, beneath it, public misunderstanding of science. To correct this trend, I argue that we need to focus on teaching how science works rather than teaching scientific knowledge or facts.

## 1. Evidence doesn’t support a pandemic paradox

“Empirical data do not support the conclusion of a crisis of public trust in science,” say Naomi Oreskes and Erik M. Conway (2022) but, they continue, “do support the conclusion of a crisis of conservative trust in science” (p. 98). A very high-powered survey of >70,000 respondents in 68 countries by Viktoria Cologna et al. (2025) found that trust in science is widespread and only tapers among conservatives. This trend has been identified by lots of social scientists, including Rainer Bromme et al. (2022), Roderick Rekker (2025), Manjana Milkoreit and E. Keith Smith (2025).

Not only is there no crisis of *public* trust in science, recent evidence suggests that there’s no *crisis* either because trust in institutions has been declining for decades. Seeking to test whether there’s a “crisis of political trust” in institutions, Viktor Valgarðsson et al. (2025) analysed a dataset constituted by over 5 million respondents in 143 countries over six decades. They found that trust in “representative institutions” (e.g. parliament, government) has generally been declining, whereas trust in “implementing institutions” (e.g. civil service, legal system) has been stable or rising. Similarly, sociologists who’ve analysed decades of data from the General Social Survey have found that conservatives and churchgoers tend to trust science less. These include Gordon Gauchat (2012), John H. Evans (2013), Darren E. Sherkat (2017), Marcus Mann and Cyrus Schleifer (2020) and Timothy O’Brien and Shiri Noy (2020).

Where does that leave the pandemic paradox? It’s not a paradox because decreases in trust are contextual, not paradoxical; nor is it a unique pandemic crisis because these are long-term trends. So, in short, there is no pandemic paradox. However, there is a long-term polarization of trust in science. This was exacerbated by the pandemic: Sofia Radrizzani et al. (2023) found that a major predictor of post-pandemic decreases in trust was low pre-pandemic trust.

## 2. Trust in science is polarizing over time

The long-term decrease in trust in science among conservatives is increasingly attributed to political bias. Perhaps because a lot less research has been done on increases in trust in science, it’s often believed (or implied) that the biasing effect of political beliefs on trust in science is asymmetrical: namely, liberals and moderates trust science to the extent that it’s trustworthy, while conservatives are biased and distrust science. However, I theorize that, as well as a conservative loss of trust in science, there’s a corresponding liberal gain in trust in science. I argue that neither liberal nor conservative trust in science corresponds to the actual trustworthiness of science.

One resource to draw on for evidence of the symmetry of political bias is the literature on conspiracy theories. It used to be thought that conservatives are more prone to conspiratorial thinking, but more recent evidence collected by Roland Imhoff et al. (2022) shows that radical liberals and conservatives are both prone to conspiracy theorizing, which Yijia Erika Zhu and Sebastian Scherr (2025) describe as “hook-shaped polarization” of conspiracy theories. Steven Smallpage et al. (2017) suggest that many conspiracy theories have bipartisan support and Joseph Uscinski et al. (2016, 2021) find that a conspiratorial predisposition is balanced between the right and left. As Adam Enders et al. (2023) put it, “for every investigation demonstrating asymmetry there is a counterweight showing the opposite” (p. 2004).

This evidence partly feeds into the “horseshoe theory” narrative which maintains that political extremes meet. There’s been little work on this, and many political scientists like Alain Van Hiel (2012) believe it to have been discredited. However, there are certain aspects of horseshoe theory that enjoy empirical support. For example, Herbert McClosky and Dennis Chong (1985) found that extreme liberals and conservatives both see society as dominated by conspiratorial forces. More recently, Eduardo Ryô Tamaki and Yujin J. Jung (2025) also found that political ideology and populist attitudes exhibit a U-shaped nonlinear relationship. I don’t draw on the specific horseshoe theoretic claim that extreme right and left are *alike* – my claim is that they’re both prone to political bias and that this same mechanism can result in too much liberal trust in science and too little conservative trust.

Three studies directly support my specific claim about the bilateral politicization of trust in science. First, Floyd Jiuyun Zhang (2023) found that conservatives lost trust in science as a result of the endorsement of political candidates by Nature (2020). This was the headline finding of the article and has attracted a lot of attention, but this study also found that liberal trust actually *increased* with the political endorsement of the left-wing candidate. Second, John J. Lee (2021) reinterpreted the General Social Survey data and concluded that a significant share of the growth in polarization is due to an increase in trust in science among liberals. Third, Marlene Sophie Altenmüller and Laura Amelie Poppe (2025) observe that everyone is equally susceptible to motivated science reception (though, admittedly, their variables are character traits, not political beliefs, making this the weakest evidence of the three).

I therefore model the politicization of science in the following way (Figure 1): proximity to political extremism correlates with increasing divergence of public trust from the trustworthiness of science; liberalism is positively correlated while conservatism is negatively correlated; moderate trust in science most closely maps onto how trustworthy science actually is; and the divergence of trust from trustworthiness is caused by political bias.

**Figure 1.**
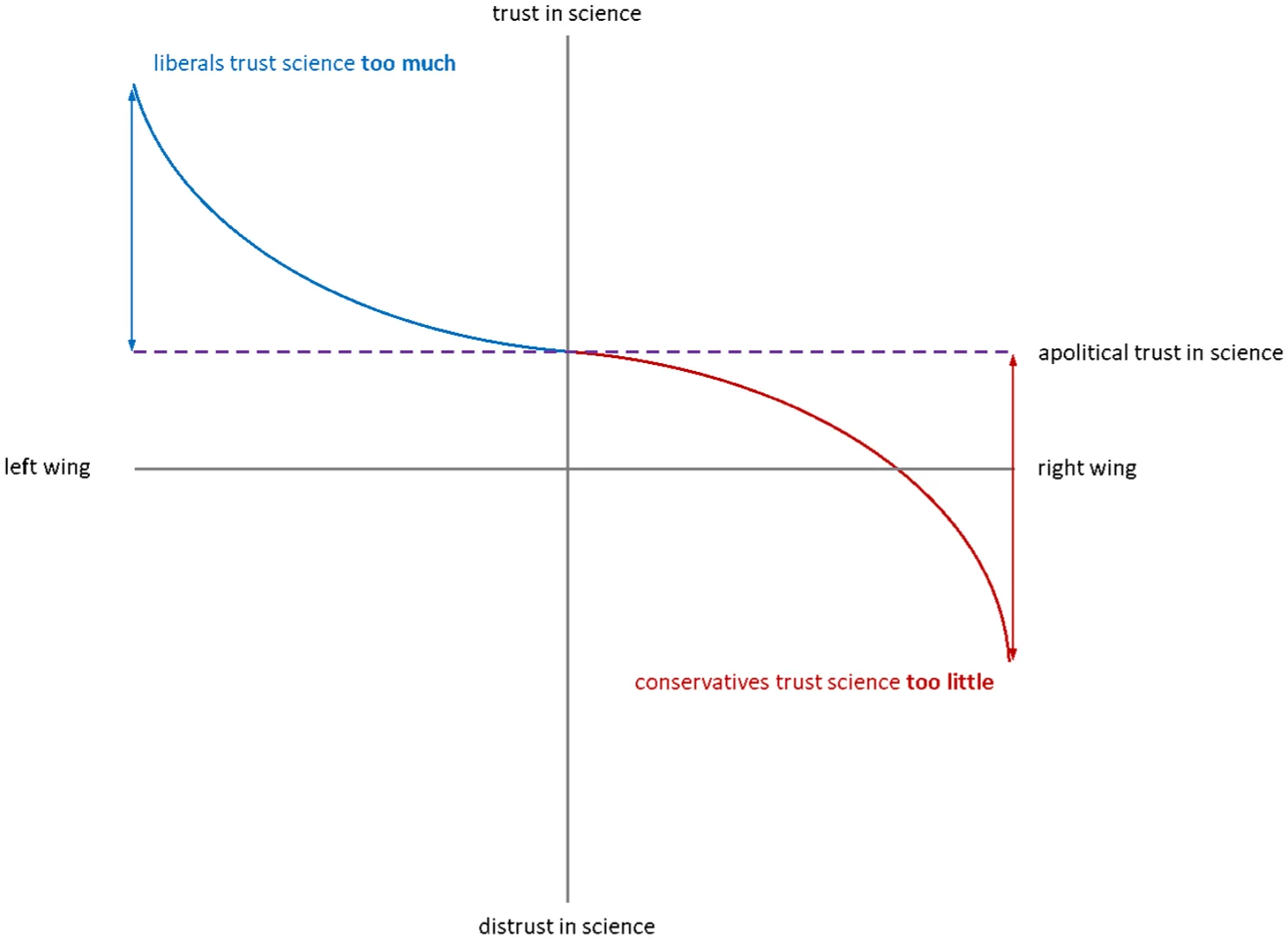
Model of the politicization of trust in science.

## 3. Public understanding determines trust

Now let’s look into the polarization of trust in science more closely. I argue that political bias isn’t the root cause. Rather, public misunderstanding of science produces an epistemic gap between public trust in science and the trustworthiness of science (Hyde, 2025). This gap is then exploited by political bias.

Most people have what Gabriele Contessa (2023) calls a “naïve view of science” where they don’t understand that science is fallible and doesn’t “prove” anything. In fact, Coosje Veldkamp et al. (2017) have shown that even scientists believe in the “storybook image of the scientist” and think too highly of themselves. This causes an epistemic gap to form between trust and trustworthiness. Often, the direction of this gap is such that, for the “median truster” (slap bang on the y axis), trust is higher than trustworthiness: the public expects too much of science, like a proof or a cure (Hyde, 2026). When the connection between these two variables weakens, it allows trust in science to be influenced by factors unrelated to trustworthiness.

The Centers for Medicare & Medicaid Services (2013) mandated that physicians disclose conflicts of interest to their patients, which Genevieve P. Kanter et al. (2019) have associated with a decrease in trust in science. This is because patients often expect science to be free from conflicts of interest, failing to appreciate that many conflicts of interest are ineliminable, and mistakenly believing that conflicts of interest are automatically evidence of funding bias. By contrast, Sunita Sah et al. (2016) have found that patients sometimes mistake disclosure of conflicts of interest for an indication of honesty or expertise (i.e. trustworthiness) and thus their trust increases. This “transparency paradox” is unlike the pandemic paradox to the extent that it can’t be explained by political bias. Instead, it’s explained by a gap between public expectations and the realities of science.

I argue that public misunderstanding of science creates space for ideological bias because epistemic gaps are necessary conditions for motivated reasoning to take hold. Wishful thinking and confirmation bias can only influence beliefs when those beliefs appear plausible. Bias couldn’t influence your trust in mathematical certainties (such as the sum of two and two equalling four) because their truth is obvious to you. Similarly, motivated reasoning about scientific claims is only possible when the scientific landscape appears contestable enough to accommodate competing interpretations. The public’s misunderstanding of science creates exactly this kind of epistemic terrain, making space for ideology to shape which scientific claims individuals find credible (Figure 2).

**Figure 2.**
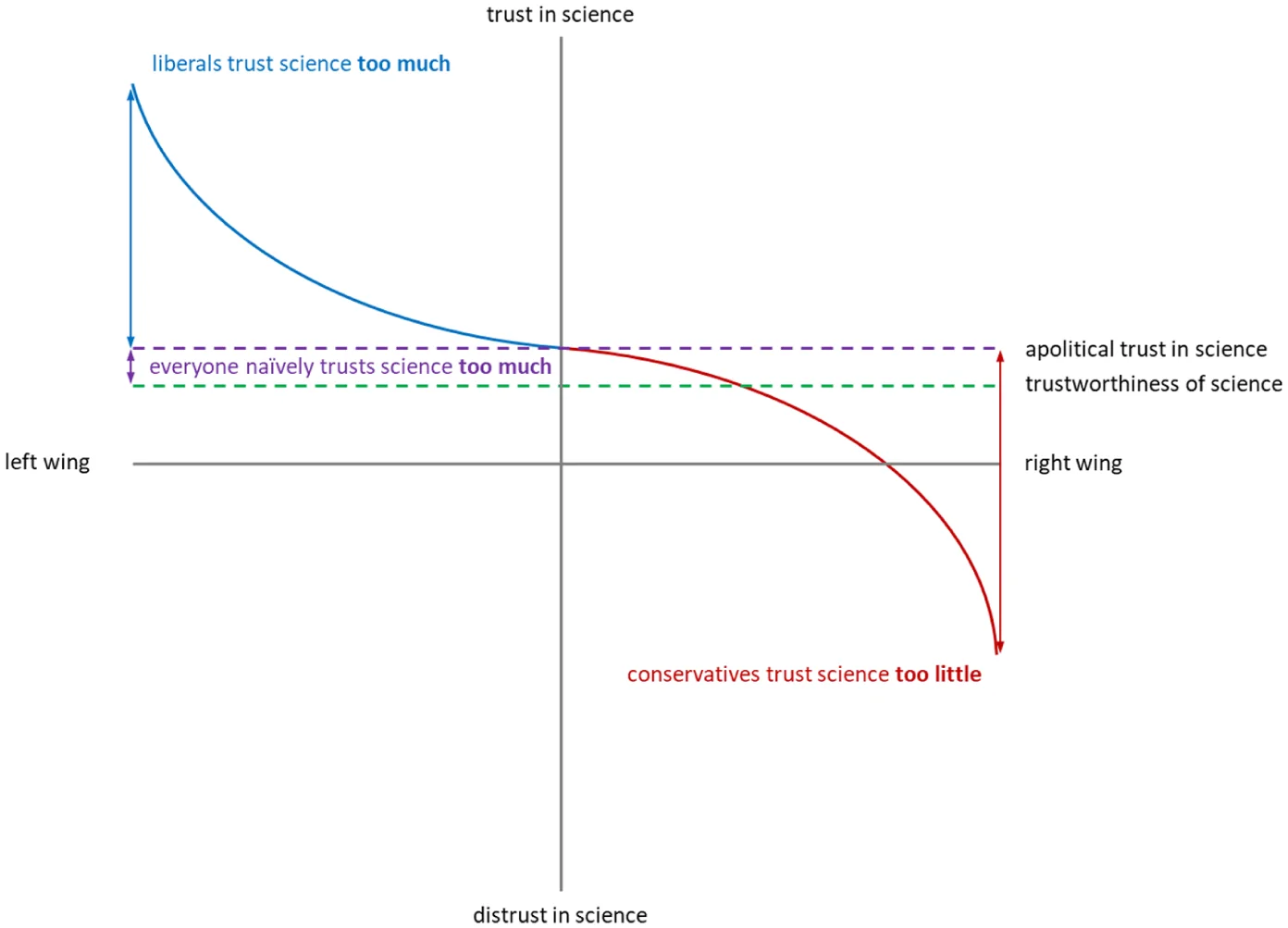
The relationship between trust in and the trustworthiness of science.

For example, Rod Abhari and Emőke-Ágnes Horvát (2025) find that article retractions can be selectively used as evidence of institutional corruption. In my model, this bias is only possible because the public often doesn’t understand that a retraction can be a positive correction of the scientific record due to the identification of an honest mistake. Radical anti-science conservatives are only able to cherry-pick evidence (i.e. retractions) because they mistakenly believe that retractions *are evidence* for corruption, which is why favourable retractions are highlighted and unfavourable ones are swept under the rug.

If public misunderstanding of science underwrites the polarization of trust in science, it would follow that, to fix the bilateral crisis of trust in science, we should focus on strengthening public understanding of science. This decreases the epistemic gap between trust and trustworthiness which can’t therefore be exploited by biases. Understanding makes the public resistant to political bias, stabilizing trust across the political spectrum. Referring back to my visual model (Figure 3), understanding both pulls the curve down (by eliminating the naïve view of science) and flattens it (by reducing the strength of the effect of political bias).

**Figure 3.**
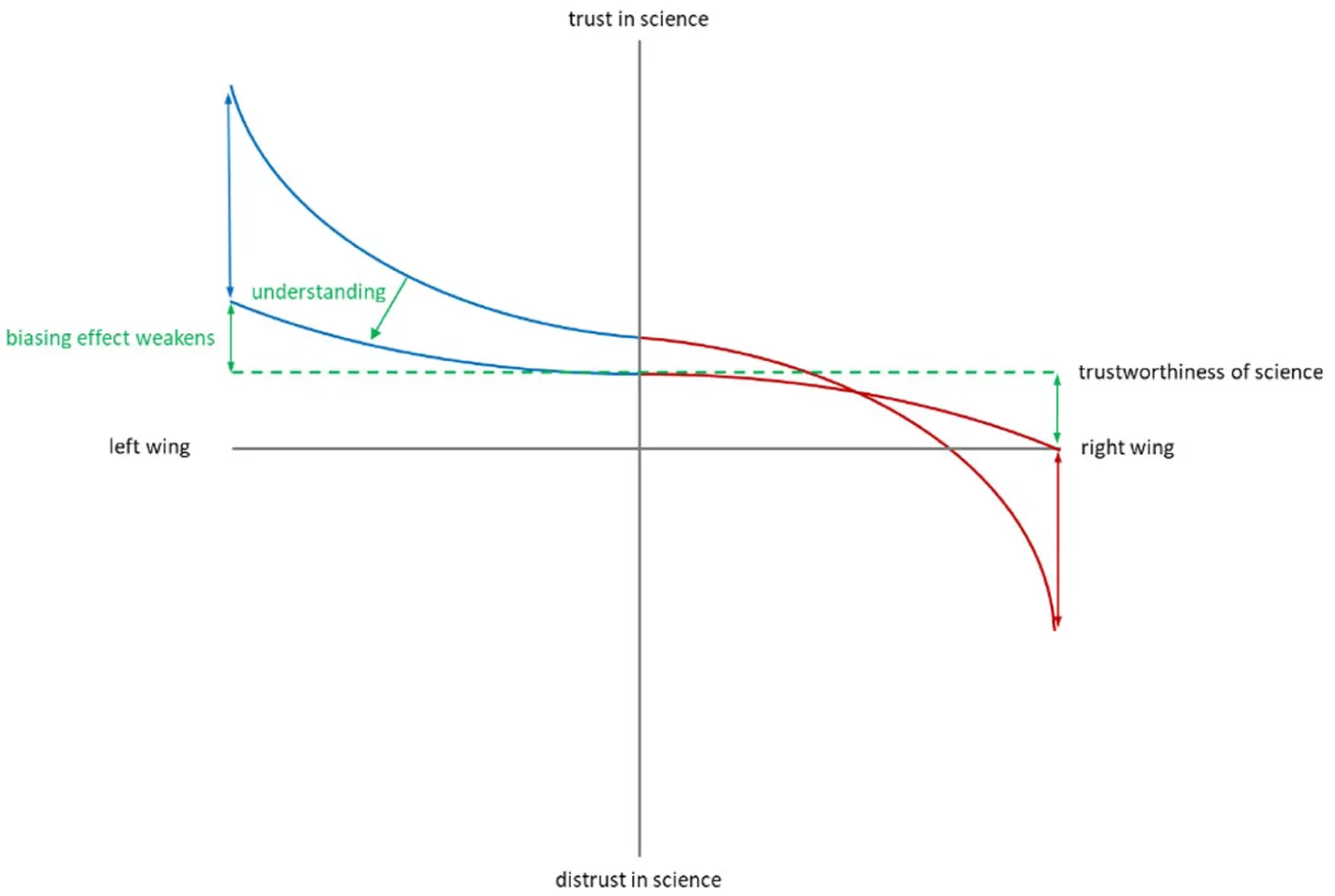
Effects of increased understanding on politicized trust in science.

Currently, there’s little evidence for my model and you won’t find that curve in any empirical articles (not least because there are other factors involved that mean it wouldn’t be nearly as symmetrical as I’ve illustrated it). However, I’m not going totally out on a limb. In the recent Research!America (2025) survey that observed an increase in trust in science, 56% of respondents said that the pandemic increased their understanding of science too. Furthermore, Michael Sailer et al. (2022) found that general knowledge of science helped individuals convert information into relevant knowledge about the coronavirus during the pandemic, which then helped them avoid panic behaviour. This might imply that their understanding of science made them resistant to biases and misinformation – and other non-trustworthiness-related factors – as I’ve theorized here.

## 4. Teach the public how science works

I argue that the public needs to understand science better. To conclude, I want to distinguish this from two common proposals intended to solve the (conservative) crisis of trust in science, both of which I argue are deficient according to my model.

It’s often argued by philosophers and scientists of science communication like Kathleen Hall Jamieson (2018) and Kristen Intemann (2023) that trust in science can be increased with communicative techniques. This might work to some extent: Rebecca Brown and Mícheál de Barra (2023a, 2023b) observe that very little public health communication mentions uncertainty, and Chelsea Ratcliff and Rebekah Wicke (2023) provide empirical evidence that doing so increases trust in science. However, this isn’t a complete solution because people don’t understand how science works in the first place. There’s a more fundamental problem here that requires more than just a reminder: the public needs education, not just communication.

Many agree that more science education is needed, rooted in the wide recognition that, as reviews by psychologists like Aart Van Stekelenburg (2026) show, more educated people tend to trust science more because science literacy improves the acceptance of scientific facts. However, Nick Allum et al. (2008) have found that highly educated conservatives are *more* likely than their less educated counterparts to distrust climate science. Education has therefore been shown to be insufficient to offset the effects of political bias. The dominant “deficit model” of science communication assumes that more knowledge of science will eliminate public distrust. Its opponents, such as Patrick Sturgis and Nick Allum (2004), recognize knowledge as a determinant of public trust in science, but observe that public distrust cannot be reduced to ignorance and that the nature of the deficit is more complex than a linear relationship between knowledge and trust.

I join opponents of the deficit model to the extent that I argue political bias causes distrust in science; ignorance is just the facilitator. However, I uphold the deficit model to the extent that, as Carina Cortassa (2016) observes of advocates of the orthodoxy, I’m an optimist about the relationship between science and society. I assume that the public will trust rationally when they understand science. I assume, like philosophers of science such as Torsten Wilholt (2013) and Benjamin McCraw (2015), that “epistemic trust” – where trust and trustworthiness align – is possible.

The problem, in my optimistic view, is that science education tends to focus on knowledge or facts. Most people learn that the dinosaurs were wiped out by an asteroid, but few know *how* we know that. The few basic experiments done in school don’t teach how complex theory development works. Putting a conservative through an engineering degree isn’t going to make them any more understanding about consensuses around climate science if all they’re taught on their degree is facts about engineering. Nor do the public understand the institutional functioning of science, such as the fact that all research needs to be funded by someone and thus financial interests are ubiquitous. The problem is the *type* of education, not its quantity. I’m not necessarily advocating for full-blown philosophy of science education as the likes of H. Holden Thorp (2024) have, but I am saying that the public needs to understand how science works.

For readers of this journal, this might seem like an underwhelming conclusion. I couldn’t agree more. A report of the Royal Society (1985) from no less than 40 years ago asserted that “a proper science education at school must provide the ultimate basis for an adequate understanding of science” which, it continues, “includes not just the facts of science, but also the method and its limitations” (p. 6). There’s my conclusion in a nutshell – granted, I’ve grounded it in trust in science in a way that the Royal Society didn’t. What I’m really bringing attention to in this commentary is the fact that the relationship between science and society has always been broken and too few steps have been taken to fix it. Even now, proposed measures are too modest. If there’s one gift that the pandemic bestowed upon science, it’s the cracks it revealed under the pressure of a crisis. We now have an opportunity to learn from our mistakes and can start climbing our way out of the hole we’ve been digging for decades.
